# Supplementary material for: Accurate and reproducible enumeration of CD4 T cell counts and Hemoglobin levels using a point of care system: Comparison with conventional laboratory based testing systems in a clinical reference laboratory in Cameroon
Source: PLoS One. 2024 Mar 20;19(3):e0297790. doi: 10.1371/journal.pone.0297790 (PMC10954178; doi:10.1371/journal.pone.0297790)
Supplement: Attachment — Submitted filename: Review notes for manuscript entitled.docx [file pone.0297790.s002.docx]

Review notes for manuscript entitled: ACCURATE AND REPRODUCIBLE ENUMERATION OF CD4 T CELL COUNTS AND HEMOGLOBIN LEVELS USING A POINT OF CARE SYSTEM: COMPARISONWITH CONVENTIONAL LABORATORY BASED TESTING SYSTEMS IN A

CLINICAL REFERENCE LABORATORY IN CAMEROON

Thank you for presenting this methods comparison paper for review. Broadly, may I start by pointing out that the manuscript that has significant gaps that need to be addressed. There is no data on advantages or usability and yet conclusions have been drawn on usability. There is no data on interoperator variability even though this data was collected in the workflow. There are language barrier problems as well. The results section is incomplete.

The title in the abstract page has a mistake. “Raccurate” should probably be “Accurate”.

Abstract page 7^th^ row entitled “Abstract”, Background section, delete “information about”.

In the whole document, TM should be in superscript.

Although the background suggests that the checked the advantages of using the POC, the manuscript simply compares the performance of the FACSPresto with FACSCanto where CD4 measurements are taken, and with SYSMEX where Hb measurements are taken. Advantages such as cost, time to result, usability, breakdowns etc are not evaluated.

In the results section of the abstract, in the last sentence. I am not convinced that you demonstrated ease of use. For instance, how many steps or preanalytical procedures and analytic procedures were needed to conduct a test?

In the Ethics Statement please replace “routine services CD4 testing” with “routine CD4 testing services”.

Face Page of the manuscript: I feel as though “Vergata” should be followed by a comma and a space before “Rome”.

Overall, the manuscript can benefit from a text editing attempt, to check grammar and syntax.

In the introduction section, second paragraph. “Assess the” … delete the article “the”.

…blood. [5]. Delete the first full stop.

Third paragraph introduction section” “…characteristics like point…” Replace “like’ with “such as”.

This manuscript can benefit from line numbering and page numbering.

Paragraph 4: “…parameters of research and…” should become “…parameters for research and…”

The last sentence in paragraph 4 should be rewritten into two sentences.

In the ‘Materials and Methods’ section, the second sentence is long and unclear and should be revised.

In the samples section, the phrase “…or not processed as per protocol” has an unknown meaning. What or how do you mean when you say that?

‘Analysis on FACSCanto II” is a new subsection and not a continuation of the previous subsection.

Change the case of BD FACSCANTO to BD FACSCanto in the same section.

“…external quality assurance program with the Cameroon…” becomes “… an external quality assurance program with the Cameroon…”

In the Ethical Review section, how did you actually take the informed consent for remnant samples? Had they already been deidentified? Did you go back to the clients?

In the Results section:

“For this study for method comparison…” should be “For this methods comparison study…”

In the Deming Regression paragraph, R2 should be R^2^

Actually I have an opinion about the results section. I am familiar with describing results carefully and identifying the main findings in a paragraph, after which a figure or a table is introduced. This allows a reader to go through a results section with significant understanding before going to a table or figure. The results section of this manuscript hardly describes the data. Is there a reason for that?

The last sentence in the results section is confusing. What do you mean by “SD of the relative deviation?”.

The first sentence of the Discussions section should be rewritten. There have been some claims in this manuscript about BD FACSCanto having “validated clinical software”. Could you introduce a reference for that?

In the sentence that has reference 17, “…CD4 enumeration still…” should be “…CD4 enumeration is still…”. As a matter of fact, the whole of that sentence should be revised. The sentence that starts with “In addition…” should be rewritten as it is incomplete.

“CD4 results contribute to the interpretation of genotyping…” Why do you think so, or how do you know that? Do you have references you could use to support that?

“The BD FACSPresto results were reproduced when operated by trained operators (but not laboratory technical experts)”. Now, this statement has far-reaching ramifications. Did you speak out of turn? I should like to believe that lab experts are even more likely to find it easy to use.

For the sentence that starts with “The analyser provides absolute…” Please revise the whole sentence to read as follows: “The analyser provides absolute and percentage CD4+ T Cell countsnas well as Hb levels [15] which are all useful for monitoring the paediatric population and tracking anaemia in pregnancy. This instrument has a battery and can run without mains power for about six hours.”

The sentence after that is problematic. It says that The POC device is easy to use also for personnel not working in the lab. Where would those personnel be working? In your study, you did not evaluate that particular capability, and I would be happy to hear why you think so.

“…the loss of followup of patients..” should be “…loss to follow up…”

“…cross training lab staff and representing…” should become “…cross training lab staff and represents…”

“…facing the accreditation process…” Replace “facing” with “undergoing”.

In tha Third last paragraph in Discussions. You suggest in the second last sentence that the BD FACSPresto is simpler to use than BD FACSCanto. Clarify that.

The last paragraph needs to be rewritten, and the last 6 words should be deleted.

The figures are very confusing. Figure 2 is presented before Figure 1. And, Figure two has graphs 1A, 1B and 1C. How is that? And then Figure 3 has graphs 2A, 2b and 2C.

In Figure 1, the last process in the workflow is “Variability between Instrument/Operator, between lot..” These data are not presented in the paper at all.

In Table 1, there are several coordinates in brackets. For example the last line has 20(32, 79). What are those numbers? This issue can also be see in Table 2.
