# Supplementary material for: Accurate and reproducible enumeration of CD4 T cell counts and Hemoglobin levels using a point of care system: Comparison with conventional laboratory based testing systems in a clinical reference laboratory in Cameroon
Source: PLoS One. 2024 Mar 20;19(3):e0297790. doi: 10.1371/journal.pone.0297790 (PMC10954178; doi:10.1371/journal.pone.0297790)
Supplement: Attachment — Submitted filename: RESPONSE TO REVIEWERS.docx [file pone.0297790.s003.docx]

Review notes for manuscript entitled: ACCURATE AND REPRODUCIBLE ENUMERATION OF CD4 T CELL COUNTS AND HEMOGLOBIN LEVELS USING A POINT OF CARE SYSTEM: COMPARISONWITH CONVENTIONAL LABORATORY BASED TESTING SYSTEMS IN A

CLINICAL REFERENCE LABORATORY IN CAMEROON

Thank you for presenting this methods comparison paper for review. Broadly, may I start by pointing out that the manuscript that has significant gaps that need to be addressed. There is no data on advantages or usability and yet conclusions have been drawn on usability. There is no data on interoperator variability even though this data was collected in the workflow. There are language barrier problems as well. The results section is incomplete.

Response: Thank you for your remarks/ Prof Maria SALVATO has improve the English language of the manuscript

The title in the abstract page has a mistake. “Raccurate” should probably be “Accurate”.

Abstract page 7^th^ row entitled “Abstract”, Background section, delete “information about”.

Response: Modified in the manuscript

In the whole document, TM should be in superscript.

Although the background suggests that the checked the advantages of using the POC, the manuscript simply compares the performance of the FACSPresto with FACSCanto where CD4 measurements are taken, and with SYSMEX where Hb measurements are taken. Advantages such as cost, time to result, usability, breakdowns etc are not evaluated.

Response: Thank you for your observation, we inserted it the manuscript

In the results section of the abstract, in the last sentence. I am not convinced that you demonstrated ease of use. For instance, how many steps or preanalytical procedures and analytic procedures were needed to conduct a test?

Response: Comparing both instrument, steps in the pre analytical procedure is reduced comparing to BD FACSCanto II, because you need to do the fluidic start-up, after you have to calibrate the instrument with the 7 colors set-up for clinical software. In the FACSPresto, we have to carry one drop of sample in the cartridges and capp. When you turn on the instrument, we do not need beads, you have an internal quality control done by the instrument and if it’s in the range, the instrument is ready to read samples

In the Ethics Statement please replace “routine services CD4 testing” with “routine CD4 testing services”.

Response: Modified in the manuscript

Face Page of the manuscript: I feel as though “Vergata” should be followed by a comma and a space before “Rome”.

Response: Modified in the manuscript

Overall, the manuscript can benefit from a text editing attempt, to check grammar and syntax.

In the introduction section, second paragraph. “Assess the” … delete the article “the”.

…blood. [5]. Delete the first full stop.

Response: Modified in the manuscript

Third paragraph introduction section” “…characteristics like point…” Replace “like’ with “such as”.

Response: Modified in the manuscript

This manuscript can benefit from line numbering and page numbering.

Paragraph 4: “…parameters of research and…” should become “…parameters for research and…”

Response: Modified in the manuscript

The last sentence in paragraph 4 should be rewritten into two sentences.

Modified in the manuscript

In the ‘Materials and Methods’ section, the second sentence is long and unclear and should be revised.

Modified in the manuscript

In the samples section, the phrase “…or not processed as per protocol” has an unknown meaning. What or how do you mean when you say that?

Response: Thank you for your observation. This means that, if the sample is clotted or the volume is not sufficient, it will not be processed

‘Analysis on FACSCanto II” is a new subsection and not a continuation of the previous subsection.

Response: You are wright

Change the case of BD FACSCANTO to BD FACSCanto in the same section.

Modified in the manuscript

“…external quality assurance program with the Cameroon…” becomes “… an external quality assurance program with the Cameroon…”

Modified in the manuscript

In the Ethical Review section, how did you actually take the informed consent for remnant samples? Had they already been deidentified? Did you go back to the clients?

Response: Thank you for your observation: We informed the participants who came for their routine examinations that the remnant of their sample, if they consent, will be used to carry out a comparative study between two devices. Samples received at the laboratory receive a code. It is decoded only when printing and rendering the results

In the Results section:

“For this study for method comparison…” should be “For this methods comparison study…”

Modified in the manuscript

In the Deming Regression paragraph, R2 should be R^2^

Modified in the manuscript

Actually I have an opinion about the results section. I am familiar with describing results carefully and identifying the main findings in a paragraph, after which a figure or a table is introduced. This allows a reader to go through a results section with significant understanding before going to a table or figure. The results section of this manuscript hardly describes the data. Is there a reason for that?

Response: Thank you for the observation. No there is no reason for that.

The last sentence in the results section is confusing. What do you mean by “SD of the relative deviation?”.

Response: Modified in the manuscript as standard deviation

The first sentence of the Discussions section should be rewritten. There have been some claims in this manuscript about BD FACSCanto having “validated clinical software”. Could you introduce a reference for that?

Response: I have cancelled it in the manuscript! I was confused with the validated method of CD4 enumeration multitest with another not the validated software

In the sentence that has reference 17, “…CD4 enumeration still…” should be “…CD4 enumeration is still…”. As a matter of fact, the whole of that sentence should be revised. The sentence that starts with “In addition…” should be rewritten as it is incomplete.

Response: Modified in the manuscript

“CD4 results contribute to the interpretation of genotyping…” Why do you think so, or how do you know that? Do you have references you could use to support that?

Response: In the International research centre where I’m working, we have a Virology Lab which is doing gentoping test for patients. Our country is following the WHO guidelines. In these guidelines, CD4 count testing are not necessary and there is no funds for that. CD4 count are not done in all the country except my centre and a confessional health facility. It’s demonstrate that Routinely, the CD4 level can often indicate the urgency of putting the patient on a treatment with strong antiviral activity (sometimes quadruple therapy) in order to quickly reduce viremia and restore the CD4 level.

“The BD FACSPresto results were reproduced when operated by trained operators (but not laboratory technical experts)”. Now, this statement has far-reaching ramifications. Did you speak out of turn? I should like to believe that lab experts are even more likely to find it easy to use.

Response: The non-lab personnel is still using it with a less than one day training

For the sentence that starts with “The analyser provides absolute…” Please revise the whole sentence to read as follows: “The analyser provides absolute and percentage CD4+ T Cell countsnas well as Hb levels [15] which are all useful for monitoring the paediatric population and tracking anaemia in pregnancy. This instrument has a battery and can run without mains power for about six hours.”

Response: Modified in the manuscript

The sentence after that is problematic. It says that The POC device is easy to use also for personnel not working in the lab. Where would those personnel be working? In your study, you did not evaluate that particular capability, and I would be happy to hear why you think so.

Response: In our facility, we have a Medical section unit where we receive patient. During the one day training for this evaluation, the personnel not working in the lab, was associated and they analysed some samples, repeated by the lab personnel: the correlation was more than 99%,

“…the loss of followup of patients..” should be “…loss to follow up…”

Response: Modified in the manuscript

“…cross training lab staff and representing…” should become “…cross training lab staff and represents…”

Response: Modified in the manuscript

“…facing the accreditation process…” Replace “facing” with “undergoing”.

Response: Modified in the manuscript

In tha Third last paragraph in Discussions. You suggest in the second last sentence that the BD FACSPresto is simpler to use than BD FACSCanto. Clarify that.

Response: Thank you for your observation. I inserted in the manuscript few sentences about that saying that to use the BD FACSCanto II, it’s necessary to have some skills, to have a certain level of education. As I said, we have the software (FACSCanto Clinical software) many procedures (start up, set up, shutdown, cleaning instrument, samples staining), two reagents in the samples staining (antibodies, FACSLysing solution), many solutions (FACSFlow, Rinse, Clean, Shutdown) make noise) etc. It’s also expensive

The last paragraph needs to be rewritten, and the last 6 words should be deleted.

Response: Modified in the manuscript

The figures are very confusing. Figure 2 is presented before Figure 1. And, Figure two has graphs 1A, 1B and 1C. How is that? And then Figure 3 has graphs 2A, 2b and 2C.

Response: Modified in the manuscript

In Figure 1, the last process in the workflow is “Variability between Instrument/Operator, between lot..” These data are not presented in the paper at all.

Response: Thank you for your observation. Because of the fact that there was not significant differences, this is why we estimate not necessary to talk about

In Table 1, there are several coordinates in brackets. For example the last line has 20(32, 79). What are those numbers? This issue can also be see in Table 2.

Response: Thank you very much for your remark too important for the comprehension. Some brackets are representing the percentage, others represent the range. It’s modified in the manuscript
