# Supplementary material for: Accurate and reproducible enumeration of CD4 T cell counts and Hemoglobin levels using a point of care system: Comparison with conventional laboratory based testing systems in a clinical reference laboratory in Cameroon
Source: PLoS One. 2024 Mar 20;19(3):e0297790. doi: 10.1371/journal.pone.0297790 (PMC10954178; doi:10.1371/journal.pone.0297790)
Supplement: Attachment — Submitted filename: response to Reviewer 020124.docx [file pone.0297790.s004.docx]

6. Review Comments to the Author

Please use the space provided to explain your answers to the questions above. You may also include additional comments for the author, including concerns about dual publication, research ethics, or publication ethics. (Please upload your review as an attachment if it exceeds 20,000 characters)

Reviewer #1: My suggestions have now been addressed and the manuscript is much better. I have no further comments to make.

Reviewer #2: I have reviewed the revised manuscript by Sagnia et al on the evaluation of a CD4 POC for CD4 T cell counts and hemoglobin levels in Cameroon. Unfortunately, the manuscript still requires major revisions. Despite the potentially quality work done, there were some major gaps in the data and the overall messaging was unclear.

From what I understood, the authors were comparing the Canto to the Caliber, which measures CD4 and hemoglobin, but is lab based, and the Presto, which also measures both, as a POC. Even this was somewhat confusing as they briefly introduced the assays. Given the overlap in assays, perhaps a stronger statement on the ‘why’ the comparison and clearer descriptions of the technologies, would have given the reader a better understanding on what I assume to be the purpose of such an evaluation – the need for POC instruments that can report both CD4 counts and hemoglobin levels for use at ANC settings. And that an additional quality product provides a more competitive market and healthier landscape.

There were gaps in the Results section.
- Table 1 was introduced but not discussed. Were there any comments or statements regarding the characteristics of study participants?

Response: We modified in the manuscript

- “11 samples were excluded from analysis because of instrument problems or not processed as per protocol described above.” This seems important given the authors are evaluating the instrument. What exactly were the problems with these 11 samples? What were the instrument problems? And why were the samples not processed per protocol? Are these a potential issues with the use and roll out of this instrument?

Response: 11 samples were excluded because after de test on the FACSPresto, there were no results but the code 6102 was release, which recommended to rerun the quality control and to retest de sample. This was done and after all, no results. On the FACSCanto II, for these 11 samples, the CD4 were less than 30 cells/µL. These values are out of the validated range on the FACSPresto.

- The authors state “During the analysis, we observed that for CD4 less than 350 cells/mL, the FACSPrestoTM tends to underestimate the values compared to the FACSCantoTM II.” This is an important observation and should be further discussed. Is this a true and significant observation? What then are the implications of using Presto vs Canto?

Response: We modified in the manuscript

.
- Overall, the results section was thin and could use with more explained observations that begins to let us know how these observations could impact the Cameroon CD4 program.

Response: We modified in the manuscript

The Discussion section was also thin on meaningful commentary.
- As mentioned above, there were quite a few observations that could be further discussed.
- Another example is the opening statement on the “first study done in Cameroon on the BD FACSPrestoTM performance was published comparing BD FACSPrestoTM versus PIMA, BD FACSCountTM and BD FACSCaliburTM.” What were the findings from this study? Why did the authors bring this up in the Discussion?

Response: We modified in the manuscript

- Much of what has been included in this section belongs in the Intro or Methods sections.
- Beginning with Line 2019, the authors make what is probably the ‘take home’ message: “The present study is the first published paper to demonstrate that BD FACSPrestoTM provides excellent results when compared with a BD FACSCantoTM II clinical machine, showing very good agreement between both instruments.” But because the results were so thin, it leaves the reader questioning ‘where is the data to support this?’.
Response: We modified in the manuscript

- Overall, the Discussions section needs to be rewritten. Perhaps each of the statements made by the authors could immediately be followed by a summary of the results used to draw this conclusion and then their discussion on this, with each having its own paragraph.
- Probably too much detail was put in this section describing product characteristics, but not enough to conclude “our comparison demonstrated equivalent performance between the BD FACSPrestoTM system and the standard-of-care, the BD FACSCantoTM II system, and the Sysmex 100 XN analyzer…”.

Response: Thank you for your observation. We compared the correlation between the results obtained from these two instruments for CD4 enumeration

Minor comments
- Line 222 – “This study supports that CD4 T cells measurements were not influenced by the counting method used.” What does this mean? What counting method?

Response: This means that if you test a patient on a FACSPresto or a FACSCanto II, the results will be the same.
- Line 233 – “Placing this instrument in rural areas will reduce the loss to follow-up…” Test time to result or turn-around time has not yet been mentioned.

You are right, this has to be mentioned in the manuscript
- Line 241 – “Based on internal experience,…” This is not internal experience, but a product characteristic.

You are right, it’s the characteristic of the product
- Line 253 – “This reduces the need for specimens or patients to be transported to a centralized testing facility for accurate and reliable test results (both diagnostic and monitoring of known infections).” Is CD4 being used to monitor infections (surveillance?)? Or perhaps monitoring patient treatment?

Response: In the context of HIV/AIDS, CD4 count is used to monitoring the patient treatment. We modified in the manuscript

7. PLOS authors have the option to publish the peer review history of their article ([what does this mean?](https://journals.plos.org/plosone/s/editorial-and-peer-review-process#loc-peer-review-history)). If published, this will include your full peer review and any attached files.


If you choose “no”, your identity will remain anonymous but your review may still be made public.


**Do you want your identity to be public for this peer review?** For information about this choice, including consent withdrawal, please see our [Privacy Policy](https://www.plos.org/privacy-policy).

Reviewer #1: **Yes:** Prof. Matilu Mwau

Reviewer #2: No

While revising your submission, please upload your figure files to the Preflight Analysis and Conversion Engine (PACE) digital diagnostic tool, <https://pacev2.apexcovantage.com/>. PACE helps ensure that figures meet PLOS requirements. To use PACE, you must first register as a user. Registration is free. Then, login and navigate to the UPLOAD tab, where you will find detailed instructions on how to use the tool. If you encounter any issues or have any questions when using PACE, please email PLOS at [figures@plos.org](mailto:figures@plos.org). Please note that Supporting Information files do not need this step.

Response: We used it to transform Figures
